# Supplementary material for: Epidemiological and clinical features of Panton-Valentine Leukocidin positive Staphylococcus aureus bacteremia: A case-control study
Source: PLoS One. 2022 Mar 18;17(3):e0265476. doi: 10.1371/journal.pone.0265476 (PMC8932578; doi:10.1371/journal.pone.0265476)
Supplement: S1 File — (DOCX) [file pone.0265476.s001.docx]

**SUPPLEMENTARY MATERIAL**

**Clinical Definitions**

Sepsis: Two or more of the following respiratory rate 22 or greater, altered mentation, systolic blood pressure 100 mm Hg or less.

Septic Shock: A vasopressor requirement to maintain a mean arterial pressure of 65 mm Hg or greater and serum lactate level greater than 2 mmol/L (>18 mg/dL in the absence of hypovolemia

Bacteremia: Presence of viable bacteria in blood culture(s)

Pneumonia: Clinical features of cough, fever, pleuritic chest pain, or dyspnea AND a new chest radiographic infiltrate

Endocarditis: See the Duke Criteria

Osteomyelitis: Depending on the infected site, of pain, erythema, swelling, fever, non-healing chronic wound(s) AND MRI/xray/CT findings consistent with osteomyelitis OR a diabetic patient with a positive probe-to-bone test OR blood cultures positive with a typical organism and MRI/xray/CT findings consistent with osteomyelitis

Septic arthritis: Clinical features of arthritis (ie: joint pain, swelling, warmth, erythema, effusion) AND arthrocentesis revealing WBC > 10,000 with gram stain and/or culture showing typical organism(s)

Meningitis: Clinical features of meningismus (ie: headache, fever, or neck stiffness) along with CSF data revealing pleocytosis, with or without an organism identified on gram stain, culture, or via other CSF testing (ie: VDRL, PCR)

Skin or Soft tissue infection: erythema, warmth, pain, swelling, discharge involving skin or soft tissues

Upper respiratory infection:  acute infections involving the nose, paranasal sinuses, pharynx, larynx, trachea, and bronchi

Septic bursitis: Superficial tenderness/erythema/warm/swelling over a bursa WITHOUT suspicion for skin or soft tissue infection or septic arthritis

Focal hepatic/splenic/renal abscess: Clincal features of intra-abdominal abscess (ie: abdominal pain, fever, nausea/emesis) and imaging consistent with abscess

colonization: Presence of micro-organisms detected by staining or culture or other laboratory means without clinical signs/symptoms of infection as otherwise defined
