# Supplementary material for: Epidemiological and clinical features of Panton-Valentine Leukocidin positive Staphylococcus aureus bacteremia: A case-control study
Source: PLoS One. 2022 Mar 18;17(3):e0265476. doi: 10.1371/journal.pone.0265476 (PMC8932578; doi:10.1371/journal.pone.0265476)
Supplement: Attachment — Submitted filename: Response to Reviewers draft Jan 5 2022.docx [file pone.0265476.s002.docx]

Dear Dr. Bae,

My coauthors and I would like to thank the editors and reviewers for their thoughtful review of our manuscript entitled “Epidemiological and clinical features of Panton-Valentine Leukocidin positive Staphylococcus aureus bacteremia: a case-control study”( PONE-D-21-28267). We have now made a number of revisions to the manuscript to address the reviewer’s concerns and have attached both a clean version of the revision as well as a red-lined version showing all the revisions made to the original text.

In addition, we would like to address each of the concerns raised by the reviewers.

Reviewer #1

Major comments:

• In the selection of the case/control, was the antibiotic taken into account? The (optimal) treatment for MRSA SAB and MSSA SAB are not the same. MRSA=vancomycin and MSSA=beta-lactams/oxacillin. That being said, the proportion of MRSA/MSSA in both case and control groups were unclear. This information (and statistics) should be included in the tables. This is an essential information as vancomycin has been demonstrated to be inferior in terms of clinical cure (which is the outcome in the study) than oxacillin for treating MSSA SAB.

***In our selection of controls we did not include an adjustment for antibiotic therapy given that this was a retrospective real world review and the patient’s received multiple different antibiotic regimens initially and had multiple different subsequent modifications in antibiotic regimens. We appreciate the reviewer’s comment on the importance of providing this information, and we have now added this data to table 1and to a new table providing information on the antibiotic therapy received by the patients.***

• In the US, most PVL+ strains are MRSA (ST8 USA300). Therefore, PVL- MRSA controls would have been the best choice of control group (or at least equal proportions of MRSA/MSSA). In the study 77% of the cases were MRSA and only 29% of the control was MRSA. Did all patients in both groups receive the same antibiotic treatment (vancomycin)?

***We appreciate the reviewer’s suggestion about matching cases based on whether the case isolate was either MRSA or MSSA. However, our initial study plan was to solely assess the impact of PVL alone independent of the presence or absence of the mecA gene. In addition, the predominant use of vancomycin as opposed to a beta-lactam agent for MRSA infections would potentially have led to a worse clinical outcome for patients infected with a PVL+ isolate. This was not the finding of the study, and consequently we have chosen not to reselect a new control population for the study or to restrict our analysis to the PVL-/MRSA strains in our original control population. However, we have now added a description of this issue to the discussion section of our manuscript on lines 249 – 256.***

• Tables are not intuitive. For example, table 6. How were the categories for vancomycin determined? Were these vancomycin plasma levels? If yes, units are missing and please state so. I assume that N=number of patients (e.g. for Vancomycin categories). It was unclear how the numbers in the column should add up (Total IVDU users N=30; but VAN <0.5 N=27; 1 N=38 and 2 N=5 giving a total of N=70)? Similar to outcome of infection (16+4+16 does not add up to N=30/column total). Please check your tables and numbers. Missing values and others should be included in the tables.

***In re-reviewing the data in the tables to make certain that the results have been accurately reported we found that one there was one individual included in who had***

***simultaneous PVL+ and PVL- S. aureus bloodstream isolates; and this case has now been excluded from the study*. *This change has led to minor adjustments in results.***

***Data on follow up details were removed, as were vancomycin mic values; neither of these sets of data was felt to be relevant to this analysis.***

Minor points
• Line 58: gene in italics ***This has been corrected***
• Line 62: please cite the correct papers. Papers cited did not show the increase of ST8-USA300 in the community worldwide! (e.g. PMID: 30315958, 34160741, 26884428, 26464204, 33636329, etc..) ***Reviewing literature and adjusting citations***
• Line 67: please cite the correct papers. Pneumonia PMID: 10524952, SSTI/Abscesses PMID: 25753191. ***Reviewing literature and adjusting citations***
• Line 100: bacterial genus/species in italics ***This has been corrected***
• Line 156: explain the coding of vancomycin ***Vancomycin mic no longer included in study.***
• Lines 211: length of stay, fever and other clinical parameters can only be assessed if information on the antibiotic therapy was provided (choice of antibiotic has an effect on the clinical course) ***We have incorporated a discussion of the impact of antibiotic therapy on these outcomes, as well as a table.***

Reviewer #2

In the manuscript “Epidemiological and clinical features of Panton-Valentine Leukocidin positive Staphylococcus aureus bacteremia: a case-control study” authors describe the possible differences in clinical outcomes for SAB associated with the presence and absence of the PVL exotoxin. The manuscript presents interesting data, it is well structured and the writing quality is good.

***We thank the reviewer for his comments***

Minor comments:
1. Line 39: correct percentage of MRSA in case patients ***this has been corrected***
2. Please correct the order of tables (table #6 is after the table #3, and #4 is after #6) ***this has been corrected***
3. Line 243: correct spelling of “however” ***this has been corrected***
